# Expanding harm reduction to include fentanyl urine testing: results from a pilot in rural British Columbia

**DOI:** 10.1186/s12954-018-0224-z

**Published:** 2018-04-06

**Authors:** Silvina C. Mema, Chloe Sage, Serge Popoff, Jessica Bridgeman, Deanne Taylor, Trevor Corneil

**Affiliations:** 1 Interior Health Authority, Kelowna, British Columbia Canada; 2 School of Population and Public Health, University of British Columbia, Vancouver, Canada; 3 Aids Network Outreach Support Society, Nelson, British Columbia Canada; 4 Faculty of Health and Social Development, University of British Columbia, Vancouver, Canada

## Abstract

**Background:**

Harm reduction has been at the forefront of the response to the opioid overdose public health emergency in British Columbia (BC). The unprecedented number of opioid overdose deaths in the province calls for an expansion of harm reduction services. The purpose of this study was to determine the acceptability of a fentanyl urine drug test among people who use drugs (PWUD) and explore whether testing introduced any changes in participants’ attitudes and behaviors towards their drug use.

**Methods:**

A pilot of fentanyl urine testing was implemented in partnership with an outreach harm reduction program in rural BC. Participants were PWUD who had consumed within the last 3 days prior to the test. Participants filled out a semi-structured questionnaire at the time of the test and were invited for a follow-up interview 2 to 4 weeks after the test. Urine samples were tested with BNTX Rapid Response™ fentanyl urine strip test at a detection level of 20 ng/ml norfentanyl.

**Results:**

Of the 24 participants who completed the urine test and first interview, 4 had a positive fentanyl urine test. Fifteen clients completed the second questionnaire, 10 of whom reported introducing a behavior change after testing and the remaining 5 indicated being already engaged in harm reduction practices. All four clients who tested positive completed the second questionnaire; all but one indicated adopting behaviors towards overdose prevention.

**Discussion:**

Fentanyl urine testing appealed to illicit opioid users and may have contributed to adopting behaviors towards safer drug use. A relationship of trust between tester and client seemed important for clients who expressed concerns with privacy of the urine test results. Post-consumption urine testing could complement the use of pre-consumption drug checking in the context of harm reduction services.

**Electronic supplementary material:**

The online version of this article (10.1186/s12954-018-0224-z) contains supplementary material, which is available to authorized users.

## Introduction

A public health emergency was declared in British Columbia in April 2016 due to the unprecedented number of deaths from opioid overdoses. Causality assessment suggests fentanyl as the principal driver of the overdose death epidemic. While in 2012, fentanyl was implicated in 5% of drug overdose deaths; the proportion has increased to nearly 80% in 2017 [1].

The overdose public health emergency calls to expand the suite of harm reduction services to include fentanyl drug checking and urine testing. It is plausible that in the wake of an unexpected positive fentanyl drug check or urine test, clients will adopt harm reduction practices to reduce their overdose risk.

In a recent urine screen study in British Columbia, Amlani et al. demonstrated that about a third of clients had a positive fentanyl urine test. Among those who tested positive, 73% were not aware of their fentanyl exposure [2].

Amlani et al.’s findings suggest that a substantial proportion of illicit fentanyl consumption in BC is unintentional. The authors, however, fell short of exploring whether the positive fentanyl urine test result led clients to change their attitude and/or behavior towards drug consumption. It could be hypothesized that upon the “eye opening” positive fentanyl urine test result revealing their unintentional fentanyl consumption, a client may choose to adopt a harm reduction strategy to reduce their overdose risk. Harm reduction strategies include testing a small amount of drug before using their usual dose, using under supervision, or acquiring a naloxone kit. Furthermore, the change in behavior could extend to the client’s network and influence their behavior towards illicit drug use.

The purpose of this study was to twofold: first, to determine the acceptability of fentanyl urine testing among PWUD as part of harm reduction program in rural British Columbia, and second, to explore whether clients undergoing a fentanyl urine test had any changes in attitudes and behaviors towards illicit drug use 2 to 4 weeks after testing.

## Methods

### Study design

A two-phased mixed-methods study was designed to gather information from PWUD about the acceptability and effectiveness of fentanyl urine testing as a harm reduction intervention: Phase one involved a short semi-structured questionnaire at the time of the fentanyl urine test asking age range, gender, employment status, frequency of drug use, drug of choice, preferred mode/s of consumption, and when were illicit substances last consumed. Phase two was a semi-structured face-to-face interview delivered 2 to 4 weeks after the fentanyl urine test. Phase two questions inquired about any behavior change towards illicit drug use potentially triggered by the results of fentanyl urine testing [3–5].

All information collected was anonymous and self-reported. An acronym was used to link phase one and phase two surveys. The semi-structured interview scripts were developed based on an interview script from a provincial fentanyl and drug testing study [6] and amended by the Aids Network Outreach Support Society (ANKORS) Hepatitis C Project coordinator to reflect local language and context.

The qualitative data were analyzed using content analysis and conducted separately by three of the researchers and then discussed as a group using consensus coding techniques [7]. Final themes were presented to a small group of harm reduction experts who had contextual knowledge of the Interior region and harm reduction content expertise as a form of member checking [8].

### Participants

The pilot was promoted via word of mouth and attracted mainly long-term clients who have a relationship of trust with local harm reduction services. Occasional and frequent illicit drug users were recruited from among clients of the mobile and fixed site harm reduction program offered by ANKORS which delivers harm reduction supply in West Kootenay Communities: Nelson, Castlegar, Trail, Salmo, Fruitvale, and Grandforks. ANKORS clients were made aware of the dates, times, and locations of fentanyl urine testing opportunities through word of mouth. Clients interested in participating were informed of the study purpose, risks, and benefits, and were given the opportunity to ask questions before a team member asked for consent.

Participation in the research study was not a condition to access fentanyl urine testing. To be eligible to participate in this study, clients had to be 19 years of age or older, able to provide informed consent, and self-identify as illicit drug user. Given that fentanyl levels become negligible after 3 days, only clients who reported using drugs within this time frame were included. Fentanyl urine testing was available between March 2017 and May 2017. A $10 cash incentive was provided to study participants in two allotments of $5 the day of the fentanyl urine test and the second $5 at the end of the second visit 2 to 3 weeks later.

### Urine testing protocol

After giving consent to participate, clients were provided with sterile urine containers and asked to provide a urine sample in a private location (i.e., washroom). Once obtained, samples were left to cool to room temperature while the client was being interviewed. Samples were tested with BNTX Rapid Response™ fentanyl urine strip test at a detection level of 20 ng/ml norfentanyl. The strips were placed into the urine for 15 s, and then let sit for 5 min before reading the result to the client. As soon as the fentanyl urine test was complete, urine samples were disposed of in a toilet and flushed. Clients were then invited to return for the follow-up interview in 2 weeks.

### Ethics approval and consent to participate

Harmonized research ethics approval was obtained from the Interior Health and University of British Columbia Ethics Review boards (Board of Record Approval Reference #: 2016-17-060-I). Consent to participate in the research study was sought from each participant who expressed interest in fentanyl urine checking. Consent to participate was obtained verbally. Using a consent script (see supporting documents), staff explained the risks and benefits of the study (see Additional file 1).

## Results

Twenty-four participants completed phase one interview and the urine test. Demographic characteristics of the clients showed that about half of participants were within the 50–59 years of age range. The sample had an even distribution of men and women. Most participants indicated Income Assistance as their major source of income. Drugs of choice were cocaine, crystal meth, methadone, fentanyl, heroin, carfentanil, marihuana, ketamine, morphine, and Percocet.

The most popular mode of consumption was injection reported by over 2/3 or respondents, followed by smoking and snorting. Only one client reported using oral drugs. In terms of frequency of use, most responded that they used daily or every few days. A few clients reported using monthly and one reported using weekly. Most participants reported using more than one drug on a weekly basis.

Four of the 24 clients enrolled in phase one had a positive fentanyl urine test. Of these four, only one individual was surprised by the positive result stating that the drugs they had done were “too weak to be fentanyl.” The remaining three participants’ responses to the positive fentanyl urine test result varied from no comment to not being surprised either because they had bought fentanyl and the test confirmed what they had purchased or because they assumed fentanyl was in “everything,” referring to all illicit drugs.

Of the 24 participants who completed phase one, 15 returned to complete the phase two. Of these, 10 reported a change in behavior after testing their urine, and the remaining 5 answered that they had not introduced any behavior change. All four clients whose urine tested positive in phase one returned for phase two, three of them reported adopting a harm reduction strategy after testing. Table 1 shows some quotes of three participants who had a positive urine test result and reported introducing a behavior change. Of the 5 individuals that answered not introducing any behavior change in phase two interview, all indicated that they were already engaged in harm reduction practices. Table 2 outlines some of the answers of the 15 participants who completed phase two, regardless of the positive or negative test result.

**Table 1:** Responses given by clients who tested positive and introduced a behavior change

Any behavior changes since receiving positive results?	
Made me more careful.	
Completely avoid fentanyl.	
I did not do the fentanyl we tested	
I don’t use alone and always carry Naloxone	
I don’t use alone and injecting less now.

**Table 2:** Behavior change as reported by the 15 participants in phase two

Any changes in drug use?	Comment
No	But got some naloxone
	But have more awareness to test my stuff
	But I have been using less b/c out of money and no supply
	Dope is really weak here and I don’t use alone
	I only use pharm morphine and I won’t buy anything else
	Test was negative and always careful
	Usually use alone. I use morphine cause I know what I’m getting
	Carry naloxone kit and worry all the time
	Dope is really weak here and I don’t use alone
Yes	Did it one more time. Starting treatment
	Didn’t do the fentanyl we tested. Don’t use alone and injecting less
	I haven’t done any drugs since. I got scared. I have a drug counsellor now.
	Yes - made me more careful. Completely avoid fentanyl
	More careful about what I buy. But still use b/c I still have same problems
	Slowed my use

There were mixed results about whether expanding drug checking and urine testing services would be useful. Most participants were of the opinion such that fentanyl drug checking and urine testing services would be valuable as long as fentanyl analogues were included. Stigma was mentioned as a potential barrier to accessing services, many of the respondents indicated that they would either use this service in the privacy of their homes and/or from trusted harm reduction agency workers as evidenced by the following clients’ statements: “If you are looking for help, have more [help] available and less stigmatizing to get help there.” “Don’t feel comfortable going anywhere else but in my home or with [names harm reduction agency worker].”

## Discussion

The objective of this study was to determine acceptability of fentanyl urine testing among PWUD in rural BC and determine any behavior changes introduced in the 2 weeks after testing. The pilot project aimed to enhance outreach harm reduction services in smaller communities in the context of a provincial overdose public health emergency. It was hypothesized that a fentanyl drug testing program may be beneficial to PWUD in rural communities by attracting people who would otherwise not connect to harm reduction services, lead to behavior change through a meaningful interaction with staff, and normalize the conversation around drug use, and potentially lead to reducing stigma.

Although preliminary, our results suggest that there is a demand for fentanyl urine testing and provide an evidence base to support expansion of harm reduction services to include this service within the scope of harm reduction services. Of note, during the study period a police notification was released that carfentanil had been detected in the community which may have increased interest in testing among clients, even though the sensitivity and specificity of the fentanyl urine strips to detect carfentanil is uncertain.

It is important to differentiate between post-consumption urine testing and pre-consumption drug checking [9]. Clients indicated that their preference for checking drugs *before* consumption as opposed to getting a urine test after consumption. Drug checking for fentanyl may also positively impact behavior change leading to a decrease in overdose incidence, as suggested by a recent evaluation of a fentanyl drug checking program in a supervised consumption setting [10].

Although generally acceptable, clients expressed that they would use a urine testing service as long as privacy was maintained and the test was delivered by a trusted person. During this study, some clients expressed concerns around privacy and questioned whether the fentanyl urine test would become part of their medical record. They only agreed to test after being reassured that participation in the study was anonymous. This suggests that a fentanyl urine test program may only appeal to clients with a trusted relationship with harm reduction providers such as ANKORS workers who have delivered services in the area for over 20 years. Allowing users to test themselves may be the way around this. However, the interaction with a harm reduction worker may create a "teachable moment" critical in effecting any behavior change.

The number of positive tests was approximately 21%, slightly lower than expected given the evidence from Amlani et al.’s study which detected 29% positivity. Furthermore, wide media coverage of the overdose emergency has led to clients being aware that fentanyl is ubiquitous in the illicit drug market. The positive result was unexpected to only one of the four, compared to most of participants in Amlani et al.’s study. Interestingly, a handful of clients were expecting a positive fentanyl test result but tested negative, which could be due to a limitation of the test in detecting some of the fentanyl analogues that may be in circulation in the illicit market.

Our results suggest that the impact of fentanyl urine testing on behavior change is promising. Drawing from health promotion constructs of behavior change [11], we hypothesize that a positive fentanyl urine test result will likely increase clients’ perception of susceptibility of being exposed to fentanyl because a positive urine test result confirms fentanyl exposure almost unequivocally. We further hypothesize that changes in perceived susceptibility may extend beyond clients who test to other peers who use with them or share the same dealer as they become aware of the positive test through word of mouth. Increased susceptibility by itself will not lead to behavior change unless clients’ *believe in the benefits* of changing behavior (such as using less or not at all, using a buddy system, and carrying naloxone) and any perceived barriers to taking action are overcome. The role of harm reduction is to activate readiness to change among clients that, due to the positive test, feel susceptible to a fentanyl overdose. By providing awareness and support, this pragmatic approach increases clients’ confidence in their ability to take action, allowing clients to take control over their health to ultimately reduce their risk of a fatal overdose.

These results should be interpreted with caution given that, as with other screening programs, it is possible that fentanyl urine testing appeals to a health conscious, potentially more experienced population of people who use drugs (PWUD). Clients included in this study were already engaged in harm reduction practices through ANKORS services over several years in which may have underestimated the effect of the intervention on harm reduction uptake because they were already positively influencing behavior among clients in these areas. In addition, we could not determine whether any changes introduced by the fentanyl drug testing were sustained beyond the study period. Future studies should use a larger sample size to investigate if and if so how, these changes may be sustained.

Limitations of this study include small sample size, and the fentanyl urine test limitations to detect analogues of fentanyl and an inability to detect fentanyl beyond the 3 days due to renal clearance. It could be argued that in self-reporting behavior clients could have exaggerated any changes to please the interviewer, overestimating the impact of the intervention. To overcome this bias, interviews were carried out by harm reduction providers with long standing relationship of trust with clients. We acknowledge these limitations and believe that they do not invalidate the results of this study in exploring acceptability and demand for fentanyl urine testing.

In summary, this study suggests that fentanyl urine testing is appealing to PWUD and that it may promote behavior change towards adoption or maintenance of harm reduction strategies among PWUD in rural BC. Further research should examine whether urine testing and drug checking services may support the increased uptake of harm reduction behaviors among different groups of people who use illicit drugs.

## Additional file

Additional file 1: Consent script. (DOCX 16 kb)
