# Supplementary material for: Expanding harm reduction to include fentanyl urine testing: results from a pilot in rural British Columbia
Source: Harm Reduct J. 2018 Apr 6;15:19. doi: 10.1186/s12954-018-0224-z (PMC5889598; doi:10.1186/s12954-018-0224-z)
Supplement: Supplementary file 1 — Consent script. (DOCX 16 kb) [file 12954_2018_224_MOESM1_ESM.docx]

**Consent Script**

**Staff facilitating fentanyl urine testing:**

Please use the following script to obtain consent and cover risks/benefits at the time of the fentanyl urine testing.

**Determining eligibility**

Please ask the client whether they are currently prescribed fentanyl for medical purposes (i.e. fentanyl patch, pills)

**Time spent if you chose to participate**

Phase 1 is delivered on the day of fentanyl urine testing. It includes two semi-structured interviews plus the fentanyl test. It is anticipated that the two semi-structured interviews will take approximately 10-20 minutes to complete the two, and the urine collection, fentanyl test result may take another 10 minutes to complete. Phase one will have a duration of approximately 30 minutes.

Phase 2 consists of the semi-structured interview 2 to 3 weeks prior to phase 1. It is anticipated that phase 2 will take between 15 to 20 minutes to complete

Phases 1 and 2 combined will approximately take 50 minutes of the client’s time.

**Obtaining informed consent**

Please assess the client for their level of alert and understanding of the service being offered and exclude clients that are obviously intoxicated to a level where level understanding and judging may be impared (ie nodding off). Explain that testing will not be done at that time and instruct to return where appropriate.

Only proceed with fentanyl urine testing once you:

1. Have explained to the client and are satisfied that the client understands the nature and consequences and the reasonably foreseeable benefits and risks of fentanyl urine testing,

and

(b) Have determined that fentanyl urine testing is in the client’s best interests.

**Risk from false reassurance by a negative fentanyl urine test:**

-A negative test does not rule out that the client may have been exposed to fentanyl in the days prior to the fentanyl urine test. Fentanyl’s half-life (the time it takes to eliminate half of the dose) varies depending on mode of ingestion (ie oral, injected, transdermal). Therefore a negative test cannot confidently rule out prior exposure to fentanyl as the test may have been done “too late” to detect fentanyl.

-The test is not 100% accurate, when compared to the results highly sophisticated testing (gas chromatography/mass spectrometry), the fentanyl strip was correct at detecting fentanyl19 times out of 20. This means that every 20 urine samples that contain fentanyl, the test will miss one and falsely report the result as negative.

-A negative fentanyl urine test at this time does not guarantee that your next dose will be free of fentanyl, even if the drugs come from the same “batch” or the same dealer.

-The test strip used can only detect fentanyl and its most common metabolite, norfentanyl. It will not be able to detect any fentanyl analog.

**Harms to the drug supplier (i.e. dealer) when a client unexpectedly tests positive for fentanyl:**

There is a theoretical risk of a customer taking his anger back to the supplier. However, the benefits to the client (and even to other clients from the same supplier supplier) of knowing whether drug contains fentanyl outweigh any theoretical risks to the supplier.

Although the probability of any harm to the supplier is very low, please make sure to cover the following with the client:

-Fentanyl and carfentanyl contamination of drug samples is widespread; any drug can be contaminated with fentanyl.

-Your drug supplier may not know whether the drugs he/she is dealing contain fentanyl. Nobody can reassure you that your drugs are fentanyl free.

**Wrap up**

To all clients, but especially for those who test positive, offer counselling on how to prevent, recognize and respond to an overdose (ie don’t use alone, etc.) . Also offer a Take Home Naloxone kit.
